# Acceptance and Associated Risk Factors of Human Papillomavirus Vaccine Among Parents of Daughters in Intermediate Schools in Tabuk City, Saudi Arabia

**DOI:** 10.7759/cureus.43483

**Published:** 2023-08-14

**Authors:** Atheer M Alaamri, Alaa M Alghithi, Safa Salih, Hamza M Omer

**Affiliations:** 1 Preventive Medicine, King Salman Armed Forces Hospital, Tabuk, SAU; 2 Preventive Medicine, Ministry of Health, Tabuk, SAU

**Keywords:** saudi arabia, acceptance, knowledge, preventable cancer, vaccine, hpv, human papillomavirus

## Abstract

Background: Women in Saudi Arabia have little knowledge of cervical cancer, human papillomavirus (HPV), and its vaccine. This study assessed the acceptance, barriers, and facilitators of HPV vaccination and its associated factors among parents of daughters in intermediate schools during the academic year September 2022-June 2023 in Tabuk City, Saudi Arabia.

Purpose: The objective of this study was to evaluate the barriers and facilitators of HPV vaccination and its associated factors among parents of daughters in intermediate schools in Tabuk City, Saudi Arabia.

Methods: This was an analytical community-based cross-sectional study that targeted 947 parents of girls older than 15 in intermediate schools in Tabuk City. A structured questionnaire was used to collect data using a web-based survey.

Results: The knowledge about HPV and its vaccine in mothers was 1.627 times higher than in fathers, mainly when employed, highly educated, aged <40 years, and earning a higher income. In addition, the Saudis’ knowledge of HPV and its vaccine was 1.275 times higher than non-Saudis. The HPV vaccine acceptability among mothers was 1.259 times higher than the fathers, especially when non-employed, aged <40 years, and with higher income. The parent who knows the relationship between HPV and cervical cancer accepts the vaccine 1.794 times higher than those who ignore this relationship. On the other hand, the Saudi’s acceptability of the vaccine was 0.671 times lower than non-Saudis.

## Introduction

Human papillomavirus (HPV), a common sexually transmitted disease, is regarded to be the main reason for most cases of cancer cervix [1]. The estimated overall prevalence rates of HPV in cervicovaginal, placental, serum, amniotic fluid, and urine samples were 30.38%, 17.81%, 32.1%, 2.26%, and 25.5%, respectively. Countries in the African region were assessed to have the highest prevalence rates, while those in Europe and the Eastern Mediterranean had the lowest prevalence rates [2].

There are currently over 220 distinct types of HPV. The HPVs, double-stranded DNA viruses without an envelope that can spread and reproduce in epithelial cells in the skin and mucosa, have about 8000 base pair circular genomes [3].

The Papillomaviridae family of viruses includes HPVs, which are non-enveloped and tiny (diameter of 50-60 nm). They are double-stranded DNA viruses. Five identical pentameric L1 proteins are fused to a single L2 protein to form 72 capsomeres around the virus [4]. This protein is currently the subject of multiple HPV therapy investigations because of its capacity to self-assemble into highly immunogenic, non-infectious virus-like particles and high-affinity domains with the host that stimulate the immune response [5]. The regions of the L1 protein significantly influence the variety of HPV variations that define virulence or persistent risk for illness recurrence [6]. The HPV genome's tiny size limits the number of genes. However, due to the numerous promoters and intricate splicing patterns needed for gene expression, the overall number of encoded proteins is substantially higher [7].

In general, HPVs are divided, according to their ability to cause cancer, into low-risk and high-risk HPVs. Low-risk HPVs are mostly responsible for skin and anogenital warts, oropharyngeal cancer, and anogenital malignancies including anal, cervical, vaginal, vulvar, and penile cancers. However, the intraepithelial lesions in the cervical cavity associated with the high-risk HPV 16, 18, 31, 33, 35, 39, 45, 51, 52, 56, 58, 59, 68, 73, and 82 groups may progress to cervical cancer [8,9].

Simple columnar epithelium lines the upper cervix (endocervix), while stratified squamous epithelium lines the lower cervix (ectocervix). Many cervical cancers develop at the junction between them because of the significant likelihood of metaplastic alterations. The highest levels of metaplastic activity are correlated with the risk of HPV infection. After menopause, metaplastic training starts to decline after puberty and the first pregnancy [10].

The most common malignancy affecting women's health is cervical cancer. It is the fourth most prevalent illness in the world. In 2020, there were over 342,000 cases of cervical cancer deaths, with 90% of those deaths taking place in less developed nations [11]. Among Saudi Arabian women between the ages of 15 and 44, cervical cancer is the ninth most prevalent disease. According to current statistics, 358 women are diagnosed with cervical cancer per year, and 179 of them pass away because of the illness [12]. Moreover, HPV contributes to other disorders such as anogenital cancers [13]. However, compared to cervical cancer, the impact of these cancers on the population is minimal. In contrast to the estimated 500,000 incidences of cervical cancers worldwide, non-cervical anogenital malignancies are thought to occur at 60,000 annually [14].

Patients with HPV-related oropharyngeal squamous cell carcinoma had a 7% frequency of distant metastases [15]. HPV may contribute to bladder cancer carcinogenesis and a poorer prognosis for bladder cancer patients. Therefore, HPV vaccination is essential for everyone to receive to avoid bladder cancer, especially men [16].

An important risk factor for contracting HIV is HPV infection. This might have a scientific explanation because HPV infection causes genital inflammation, breakdown of the vaginal barrier, and an inflow of activated target T cells, all of which have been linked to an increased risk of contracting HIV [17].

Numerous studies have shown that pregnant women with HPV have a higher risk of having unfavorable outcomes. To enhance pregnancy outcomes, child survival, and health, it would be advantageous to understand the processes by which HPV adversely impacts pregnancy and take effective prophylactic measures into consideration [18].

To prevent and lessen the existing burden of HPV infection in pregnant women, behavioral and therapeutic interventions as well as immunization programs must be implemented [2]. The HPV vaccine is the most reliable method of preventing cervical cancer. WHO advises females between the ages of 9 and 14 (before sexual involvement) to receive routine HPV vaccination through national immunization programs, as well as "catch-up vaccinations" for unvaccinated individuals older than 15 who engage in high-risk behaviors and for women who are HIV-positive [19].

At least 107 nations have national HPV vaccination programs in place, including Saudi Arabia [20]. The Saudi Ministry of Health launched a program to immunize all girls aged 9 to 11 across the nation in collaboration with the Ministry of Education. The younger population depends heavily on parents and guardians to receive vaccines, and their willingness to get vaccinated will strongly affect their daughters' desire to do the same. In Saudi Arabia, parents have a poor understanding of and attitude toward the HPV vaccine. Knowing more about the HPV vaccine was highly connected with planning to receive it [12].

The HPV vaccine, pap smear, and early treatment of pre-cancer lesions are all reasonable approaches for preventing cervical cancer [21]. Cervical cancer is by far the illness linked to HPV that is most prevalent. HPV infection almost usually results in cervical cancer. Even while many HPV infections and pre-cancerous lesions cure spontaneously, all women are at risk for HPV infections turning chronic and pre-cancerous lesions turning into invasive cervical cancer. High-grade precancerous lesions, aggressive malignancy, and HPV infections can all be avoided with HPV vaccines, according to clinical studies and post-marketing surveillance [22].

The HPV vaccine is still not widely used in Saudi Arabia [23,24]. This is ascribed to several things, including a lack of awareness about HPV and its connection to cervical cancer, a lack of acceptance of the HPV vaccine, and the belief that HPV is a sensitive topic in the traditional Saudi community [25,26].

According to the study, women in Saudi Arabia's western province have little understanding of cervical cancer, HPV, and its vaccine. In the western part of Saudi Arabia, there is a need to inform women about HPV and its problems and to encourage awareness of them [27].

In this study, we tried to assess the acceptance, barriers, and facilitators of HPV vaccination and its associated factors among parents of daughters who are in intermediate schools during the academic year from September 2022 to June 2023 in Tabuk City, Saudi Arabia.

## Materials and methods

Study design and setting

This is an analytical community-based cross-sectional study. The study was conducted in Tabuk City. The regional capital of Saudi Arabia's Tabuk Region is Tabuk. It has 667,000 residents [12]. Tabuk City has 68 intermediate schools, 56 governmental schools, nine private schools, and three special education schools. In our study, special education schools were excluded. The study was approved by the Tabuk Institutional Review Board (approval number: TU-077/023/187).

Study population

The students were selected randomly from student lists from each school. After that, school principals and health mentors were contacted to send the questionnaire to the parents. All parents whose girls were enrolled in intermediate school during the academic year September 2022-June 2023 were included. Parents of girls older than 15 years old, anyone who had an adverse reaction to the HPV vaccine, yeast allergy, and patients with any moderate or severe disease were among the exclusion criteria.

Sample size

According to available data, the total number of female students in the included 65 intermediate schools was about 15,297 girls. The sample size was calculated using the OpenEpi version 3 software (www.OpenEpi.com, Centers for Disease Control and Prevention, Atlanta, Georgia, United States). n = [DEFF*Np(1-p)]/ [(d2/Z21-α/2*(N-1) +p*(1-p)]. Based on the aforementioned factors, 375 participants made up the bare minimum sample size (n). The authors increased the sample size to compensate for possible nonresponse; thus, 947 participants were enrolled. The sample size was divided proportionally based on the number of students in each school to reduce the selection bias.

Data collection tools

Data were collected by sending an electronic-based questionnaire to parents of intermediate girls’ schools in Tabuk City. This questionnaire development followed the steps to construct the Theory of Planned Behavior questionnaire. A thorough literature search was conducted to establish the content for this survey tool's inductively developed questionnaire, which examines factors that may influence vaccination-related behavior [28,29].

To overcome the language barrier, the questionnaire was translated into Arabic. The questionnaire contained demographic factors including age, nationality, gender and social status of the respondent, number of daughters, respondent level of education, employment status, and monthly income to assess the socioeconomic status. At the beginning of the study, participants were questioned about their understanding of cervical cancer and HPV as well as their compliance with childhood immunization schedules and whether their daughters had received the HPV vaccine or not. Parents were asked to explain their decision to accept or reject the HPV vaccine as well as any other justifications.

An evaluation of the face validity and internal consistency reliability of the questionnaire was done as part of pilot research with 30 participants who weren't part of the survey to ensure its reliability. For both the initial and repeat tool administrations, internal consistency reliability coefficients were estimated using Cronbach's coefficient alpha. It was discovered that the tool's median Cronbach's alphas (for the original and retest) were higher than 0.82. Additionally, test-retest reliability over a two-week period was evaluated, and Pearson product-moment correlations between the first and retest administrations were obtained. Correlation coefficients (r) >0.70 were observed (P 0.01).

Data analysis

SPSS Statistics version 26 (IBM Corp. Released 2019. IBM SPSS Statistics for Windows, Version 26.0. Armonk, NY: IBM Corp.) was used to analyze the data. The frequency, counts, percentages, mean, and SD were used to construct the descriptive statistics. The statistical significance of connections between research variables was evaluated using the chi-square. To explore the contributing elements of HPV vaccination acceptability, logistic regression analysis, odds ratios, and confidence intervals were used. Statistical significance was defined as a p-value of 0.05 or lower.

## Results

A total of 947 parents were interviewed in this study. The mean age of the respondents was 42.28 (SD±6.82) years, with a maximum and minimum age of 55 and 24 years, respectively. The majority of the parents, 462 (48.8%), were between the ages of 40 and 49. The majority were mothers 724 (76.5%), Saudi 868 (91.7%), and their daughters in the first year 392 (41.4%). The majority were married, lived with their partners 834 (88.1%), were university graduates 366 (38.6%), were unemployed 557 (58.8%), and had a monthly income of less than 5,000 SAR. The majority of participants, 381 (40.2%), and one daughter, 788 (83.2%) in the intermediate stage, completed all vaccinations according to the national immunization schedule, 890 (94.0%) (Table 1).

**Table 1:** Demographic characteristics of survey respondents SAR: Saudi Riyal

	Variable		n (%)
1	Age of the mother or the father (mean± SD= 42. 28 ±6.82Y; max=55 and min=24Y)	<30Y	13 (1.4)
		30-39Y	324 (34.2)
		40-49Y	462 (48.8)
		>49Y	148 (15.6)
2	Who filled out the questionnaire?	Mother	724 (76.5)
		Father	223 (23.5)
3	At which year in the intermediate stage is your daughter?	First-year	392 (41.4)
		Second year	305 (32.2)
		Third year	250 (26.4)
4	Nationality	Saudi	868 (91.7)
		Non-Saudi	79 (8.3)
5	Marital status	Married	834 (88.1)
		Widower	69 (7.3)
		Divorced	44 (4.6)
6	Education level	Not educated	41 (4.3)
		Primary school	71 (7.5)
		Mid school	146 (15.4)
		High school	269 (28.4)
		University graduate	366 (38.6)
		Postgraduate	54 (5.7)
7	Employment status	Employed	390 (41.2)
		Not employed	557 (58.8)
8	Family monthly income in SAR	<5,000 SAR	381 (40.2)
		5,000-10,000 SAR	319 (33.7)
		> 10,000 SAR	247 (26.1)
9	Number of your daughters in the intermediate stage	One daughter	788 (83.2)
		Two daughters	138 (14.6)
		>Two daughters	21 (2.2)
10	Has your daughter completed all her vaccinations according to the national immunization schedule?	Yes	890 (94.0)
		No	57 (6.0)

Awareness and knowledge of the HPV vaccine

The majority of the participants, 644 (68.0%), knew about the papillomavirus, its relation to cervical cancer 590 (62.3), and the effectiveness of the vaccine 617 (65.2%) in its prevention. The source of their information was mainly social media 252 (26.6%), followed by the Internet 248 (26.2%). It was found that more than one-third of the participating parents, 329 (34.7%), refused to vaccinate their daughters. Moreover, about 105 (11.1%) participating parents have vaccinated their daughters by obligation. Furthermore, 233 (24.6%) hesitated to vaccinate their daughters. The main reason 220 (23.4%) for the rejection was that their daughters were young or not married and, thus, were not exposed to infection. This is followed by the fact that they don’t trust that the vaccine is safe 177 (18.8%) or have insufficient information about immunization 94 (10.0%). On the other hand, the main reason for the parents to accept the vaccine was their trust in the health system 400 (42.2%), followed by the parents are keen on their daughters’ health 391 (41.3%) and the effectiveness of the vaccine to prevent cervical cancer 234 (24.7%) (Table 2).

**Table 2:** Awareness and knowledge of the HPV vaccine

	Variable		Response	n (%)
1	Do you know what the papillomavirus is?		Yes	644 (68.0)
			No	303 (32.0)
2	Do you know what the relationship of papillomavirus to cervical cancer is?		Yes	590 (62.3)
			No	357 (37.7)
3	Do you know that the papillomavirus vaccine is effective in preventing cervical cancer?		Yes	617 (65.2)
			No	330 (34.8)
4	What is the source of your information about papillomavirus?		Family and friends	72 (7.6)
			Social media	252 (26.6)
			Internet	248 (26.2)
			Health practitioner	120 (12.7)
			My education	19 (2.0)
			I have no information	236 (24.9)
5	Did your daughter have the papillomavirus vaccination?		Yes (according to my wish)	280 (29.6)
			Yes (I'm obliged)	105 (11.1)
			No (however, I agree to vaccinate her)	233 (24.6)
			No (I refuse to vaccinate her)	329 (34.7)
6	Why you planned/ plan to vaccinate your daughter	As it is safe		210 (22.2)
		As it is adequate for preventing cervical cancer		234 (24.7)
		As I'm keen on my daughter’s health		391 (41.3)
		As the advice of the health practitioner		178 (18.8)
		As I trust the health system		400 (42.2)
7	Why do/ did you refuse to vaccinate your daughter?	As I do not have enough information about the vaccine		94 (10.0)
		As my daughter got the coronavirus vaccine (so much for her)		76 (8.1)
		As I think that the vaccine is a trick from the drug companies		40 (4.3)
		As I do not trust that the vaccine is safe		177 (18.8)
		As the vaccine is still new		41 (4.4)
		As my daughter is still young and not married (not exposed)		220 (23.4)

Variables affecting knowledge of HPV and its vaccine

The knowledge of HPV and its vaccine was associated with other variables. Accordingly, the odds of HPV vaccine knowledge among the age of participating parents with age <40 years were 1.126 times (OR=1.126; 95% CI= 0.885-1.484; β= -0.018; P=0.909) higher as compared to those with parents aged 40 or more. Moreover, the mothers’ knowledge was 1.627 times (OR=1.627; 95% CI= 1.200-2.206; β= 0.571; P=0.002) higher than in fathers. Furthermore, the Saudis’ knowledge of HPV and its vaccine was 1.275 times (OR=1.275; 95% CI= 0.800-2.032; β= 0.117; P=0.472) higher than non-Saudis. Furthermore, the parents who live with their partners know about HPV and its vaccine 1.362 times (OR=1.362; 95% CI= 0.916-2.026; β= 0.227; P=0.286) higher than those who live without their partners. Likewise, the knowledge of the HPV and its vaccine in those who were employed was 1.350 times (OR=1.350; 95% CI= 1.031-1.768; β= 0.280; P=0.123) higher than those who were not employed. By the same token, the parents whose daughters completed all their vaccinations scheduled know about HPV and its vaccine 1.527 times (OR=1.527; 95% CI= 0.892-2.614; β= 0.296; P=0.295) higher than those who didn’t complete all their vaccinations scheduled.

On the other hand, the knowledge of parents whose education was prior to university was 0.529 times (OR=0.529; 95% CI=0.403-0.693; = -0.434; P=0.008) lower than that of university graduates or postgraduates. In addition, the parents with an income of 10,000 SAR or less know about the HPV and its vaccine 0.732 times (OR=0.732; 95% CI= 0.892-2.614; β= 0.296; P=0.295) less than those with an income of more than 10,000 SAR per month (Table 3).

**Table 3:** Odds ratio and binary logistic regression for factors associated with knowledge of the HPV vaccine SAR: Saudi Riyal, HPV: human papillomavirus ** significant at p <0.05 level

Variable		Knowledge of the HPV vaccine		OR (95%CI)	β	P
		Yes (%)	No (%)			
Age	<40Y	216 (64.1)	121 (34.9)	1.126 (0.855-1.484)	-0.018	0.909
	40Y or older	374 (61.3)	236 (38.7)			
Who answered the questionnaire	Mother	471 (65.1)	253 (34.9)	1.627 (1.200-2.206)	0.571	0.002**
	Father	119 (53.4)	104 (46.6)			
Nationality	Saudi	545 (62.8)	323 (37.2)	1.275 (0.800-2.032)	0.117	0.472
	Non-Saudi	45 (57.0)	34 (43.0)			
Whether the parents live with their partners	Lives with their partner	527 (63.2)	307 (36.8)	1.362 (0.916-2.026)	0.227	0.286
	Lives without their partner	63 (55.8)	50 (44.2)			
Educational level	Before university	294 (55.8)	233 (44.2)	0.529 (0.403-0.693)	-0.434	0.008**
	University or postgraduate	296 (70.5)	124 (29.5)			
Employment status	Employed	259 (66.4)	131 (33.6)	1.350 (1.031-1.768)	0.280	0.123
	Not employed	331 (59.4)	226 (40.6)			
Family monthly income in SAR	10,000 or less	423 (60.4)	277 (39.6)	0.732 (0.538-0.994)	-0.113	0.521
	>10,000	167 (67.6)	80 (32.4)			
Has your daughter completed all her vaccinations scheduled?	Yes	560 (62.9)	330 (37.1)	1.527 (0.892-2.614)	0.296	0.295
	No	30 (52.6)	27 (47.4)			

Variables affecting the acceptability of the HPV vaccine

Vaccine acceptability was associated with other variables. Accordingly, the odds of HPV vaccine acceptability among the age of participating parents with age <40 years were 1.375 times (OR=1.375; 95% CI= 1.037-1.824; β= -0.286; P=0.063) higher as compared to those with parents aged 40 or older. Moreover, the mothers’ acceptability was 1.259 times (OR=1.259; 95% CI= 0.925-1.715; β= 0.130; P=0.483) higher than the fathers. Furthermore, the parents who live with their partner accept the vaccine 1.117 times (OR=1.117; 95% CI= 0.744-1.675; β= 0.114; P=0.597) higher than those who live without their partners. Likewise, the parents with an income of 10,000 SAR or less accept the vaccine 1.141 times (OR=1.141; 95% CI= 0.845-1.540; β= 0.141; P=0.412) higher than those with more than 10,000 SAR per month. By the same token, the parents whose daughters completed all their vaccinations scheduled accept the vaccine 1.546 times (OR=1.546; 95% CI= 0.902-2.651; β= 0.423; P=0.133) higher than those who didn’t complete all their vaccinations. In addition, the parent who knows the relationship between HPV and cervical cancer accepts the vaccine 1.794 times (OR=1.794; 95% CI= 1.366-2.356; β= 0.575; <P=0.001) higher than those who ignore this relationship. On the other hand, the Saudi’s acceptability to the vaccine was 0.671 times (OR=0.671; 95% CI= 0.402-1.118; β= 0.434; P=0.106) lower than non-Saudi. In addition, the parents whose education was prior to university accepted the vaccine 0.940 times (OR=0.940; 95% CI= 0.719-1.228; β= 0.046; P=0.780) lower than those who were university graduates or postgraduates. Furthermore, the employed parents accepted the vaccine 0.956 times (OR=0.956; 95% CI= 0.730-1.252; β= 0.077; P=0.669) lower than those not employed (Table 4).

**Table 4:** Odds ratio and binary logistic regression for factors associated with acceptance of HPV vaccine SAR: Saudi Riyal, HPV: human papillomavirus ** Significant at p<0.05 level

Variable		Accept vaccine		OR (95%CI)	β	P
		Yes (%)	No (%)			
Age	<40Y	232 (68.8)	105 (31.2)	1.375 (1.037-1.824)	-0.286	0.063
	40Y or older	376 (61.6)	234 (38.4)			
Who answered the questionnaire	Mother	474 (65.6)	250 (34.5)	1.259 (0.925-1.715)	0.130	0.483
	Father	134 (60.1)	89 (39.9)			
Nationality	Saudi	551 (63.5)	317 (36.5)	0.671 (0.402-1.118)	-0.434	0.106
	Non-Saudi	57 (72.2)	22 (27.8)			
Whether the parents live with their partners	Lives with their partner	538 (64.5)	296 (35.5)	1.117 (0.744-1.675)	0.114	0.597
	Lives without their partner	70 (61.9)	43 (38.1)			
Educational level	Before university	335 (63.6)	192 (36.4)	0.940 (0719-1.228)	0.046	0.780
	University or postgraduate	273 (65.0)	147 (35.0)			
Employment status	Employed	248 (63.6)	142 (63.4)	0.956 (0.730-1.252)	0.077	0.669
	Not employed	360 (64.6)	197 (35.4)			
Family monthly income in SAR	10,000 or less	455 (65.0)	245 (35.0)	1.141 (0.845-1.540)	0.141	0.412
	>10,000	153 (61.9)	94 (38.1)			
Has your daughter completed all her vaccinations scheduled?	Yes	577 (64.8)	313 (35.2)	1.546 (0.902-2.651)	0.423	0.133
	No	31 (54.4)	26 (45.6)			
Do you know what is the relationship of HPV to cervical cancer?	Yes	409 (69.3)	181 (30.7)	1.794 (1.366-2.356)	0.575	<0.001**
	No	199 (55.7)	158 (44.3)			

## Discussion

To increase the breadth of vaccination, it is vital to raise public awareness of the HPV vaccine because vaccination depends on immunization. This study aimed to ascertain whether parents of daughters enrolled in intermediate schools in Tabuk City, Saudi Arabia, accepted the HPV vaccination, what obstacles they faced, and what helped them do so.

Awareness and knowledge of the HPV vaccine

In the current work, 68.0% of parents were found to be aware of the HPV vaccine. In comparison to Italy (69.9%) [30] and the USA (76.8%) [31], this is a little lower. However, it is higher than those in China (40.27%) [32], India (36.8%) [33], Turkey (26.6%) [34], and Thailand (25.3%) [35].

The source of information in the current work about HPV and its vaccination was mainly social media 26.6% and the Internet 26.2%. These findings were consistent with earlier systematic reviews that looked at how social media might affect people's awareness of HPV and its vaccination [36-38]. This is not in line with the results of Farsi et al. who found that the education of medical students in Saudi Arabia is the main source of information; however, social media and the Internet came as minor sources [39]. In addition, Lindsay et al. reported that most parents (83.3%) who took part in their study gained their information from their child's doctor [31]. These findings underline the significance of the online resources used, as noted by Lmm et al. [40], who stressed the substantial amount of false information present on anti-vaccine websites. In this regard, the right channels should be used to help increase the knowledge and acceptability of HPV vaccination and meet the objective of eradicating HPV-related diseases.

When compared to parents who were 40 or older, parents who participated in the current study had 1.126 times the odds of knowing about the HPV vaccine (OR=1.126; 95% CI= 0.885-1.484; = -0.018; P=0.909). Lopez et al., on the other hand, claimed that parents aged 30-39 years had less average knowledge of the HPV vaccine than parents over 40 years (P=0.001) [41].

The current study revealed that the mothers’ knowledge was 1.627 times (OR=1.627; 95% CI= 1.200-2.206; β= 0.571; P=0.002) higher than in fathers. These findings agree with the results of other previous studies [31,41]. This emphasizes the need to raise men's understanding of the disease.

According to the current work, Saudis knew 1.275 times more about HPV and its vaccine than non-Saudis (OR=1.275; 95% CI= 0.800-2.032; = 0.117; P=0.472). Similarly, employed people know 1.350 times more than unemployed people (OR=1.350; 95% CI= 1.031-1.768; = 0.280; P=0.123) about this disease. These findings conflict with those made by Lopez et al. who discovered that there were no differences in knowledge concerning the employment of nationality of Spain residents [41].

The current study discovered that the knowledge of parents with prior-to-university education was 0.529 times (OR=0.529; 95% CI= 0.403-0.693; = -0.434; P=0.008) lower than that of parents with postgraduate degrees or university degrees. These findings are consistent with recent studies that found that as parental education increased, there was a significant increase in the average level of understanding about HPV (P=0.001 for all variables) [41].

The current study discovered that parents with incomes of 10,000 SAR or less know 0.732 times (OR=0.732; 95% CI= 0.892-2.614; = 0.296; P=0.295) less about HPV and its vaccine than parents with incomes of more than 10,000 SAR per month. These findings were in line with those of Lindsay et al., who stated that parents with lower income had statistically significantly lower awareness of the HPV vaccine [31].

Acceptability of the HPV vaccine

In the current work, participants refused that their daughters get the HPV vaccine as they are still young or unmarried and, thus, don’t need the vaccine (23.4%) or don’t trust the vaccine (18.8%). These findings correspond to those obtained by Farsi et al., who mentioned that the leading cause for refusing the vaccine was that they were not sexually active and not in need [40].

According to the current study, acceptance of the HPV vaccine was 1.375 times greater in parents under 40 years old than in parents over 40 years old (OR=1.375; 95% CI=1.037-1.824; = -0.286; P=0.063). These findings disagree with those of another study that found older parents to have higher levels of vaccine acceptance than younger parents (p =0.028) [41].

The present study showed that the mothers’ acceptability was 1.259 times (OR=1.259; 95% CI= 0.925-1.715; β= 0.130; P=0.483) higher than the fathers. These findings are in line with those of Mortensen et al., who stated that the gender of the parent is one of the key variables which affect HPV knowledge and vaccine acceptability [42].

The current work found that parents who earn less than 10,000 SAR per month receive the vaccine 1.141 times more often than parents who earn more than that amount (OR=1.141; 95% CI= 0.845-1.540; = 0.141; P=0.412). Other studies in China confirmed our results [43,44]. On the other hand, another study posted that parents with higher incomes were more likely to put off or reject an HPV vaccination than other households [45].

The current work found that the parents whose daughters completed all their vaccinations scheduled accepted the vaccine 1.546 times (OR=1.546; 95% CI= 0.902-2.651; β= 0.423; P=0.133) higher than those who didn’t complete all their vaccinations. The same results were posted in a Poland study based on the results; the parent whose daughters completed all their vaccinations scheduled and accepted more of the HPV vaccination [46].

According to the results of the current study, parents who were aware of the connection between HPV and cervical cancer were 1.794 times more likely to accept the vaccine than parents who were oblivious of it (OR=1.794; 95% CI=1.366-2.356; =0.575; P=0.001). This was comparable to the Ethiopian study [47]. This might make it easier for parents to comprehend the advantages of immunization.

Furthermore, the parents with education prior to university accepted the vaccine 0.940 times (OR=0.940; 95% CI= 0.719-1.228; β= 0.046; P=0.780) lower than those who were university graduates or postgraduates. These findings are consistent with those of Dorell et al. (2014), who stated that parents with more education had a higher propensity than other households to delay or refuse an HPV vaccination [45]. These findings, however, contrasted with those made public by Sobierajski et al., who noted that parents with higher levels of education were nearly twice as likely to support vaccines as those with only a primary education [46].

## Conclusions

The results of this study indicate that there is somewhat of a shortage of knowledge and attitude regarding HPV and less acceptance of its vaccine. Moreover, the results emphasize the value of educational initiatives to raise public knowledge and awareness about HPV.

Various tactics should be used, including social media campaigns, educational events, and curriculum changes. Parents' decisions to vaccinate their children are also heavily influenced by the advice of a clinician.
